# Quality Improvement Project of a Massive Transfusion Protocol (MTP) to Reduce Wastage of Blood Components

**DOI:** 10.3390/ijerph18010274

**Published:** 2021-01-01

**Authors:** Matteo Paganini, Hesham Abowali, Gerardo Bosco, Maha Balouch, Garrett Enten, Jin Deng, Aryeh Shander, David Ciesla, Jason Wilson, Enrico Camporesi

**Affiliations:** 1 TEAMHealth Anesthesia, Tampa General Hospital, Tampa, FL 33606, USA; habowaly@gmail.com (H.A.); mbalouch@mail.usf.edu (M.B.); garrett_enten@teamhealth.com (G.E.); aryeh_shander@teamhealth.com (A.S.); c-jwilson@tgh.org (J.W.); Enrico_Camporesi@teamhealth.com (E.C.); 2 Emergency Medicine, Department of Medicine (DIMED), University of Padova, 35128 Padova, Italy; 3 Environmental Medicine and Physiology Laboratory, Department of Biomedical Sciences, University of Padova, 35131 Padova, Italy; 4 Morsani College of Medicine, University of South Florida, Tampa, FL 33612, USA; jindeng@health.usf.edu; 5 Department of Surgery, University of South Florida, Tampa, FL 33606, USA; dciesla@health.usf.edu

**Keywords:** blood component transfusion, massive transfusion protocol, MTP, waste, quality improvement

## Abstract

Massive transfusion protocols (MTPs) facilitate the organized delivery of blood components for traumatically injured patients. MTPs vary across institutions, and ratios of blood components can change during clinical management. As a result, significant amounts of components can be wasted. We completed a review of all MTP activations from 2015 to 2018, providing an in-depth analysis of waste in our single Level 1 trauma center. An interdepartmental group analyzed patterns of blood component wastage to guide three quality improvement initiatives. Specifically, we (1) completed a digital timeline for each MTP activation and termination, (2) improved communications between departments, and (3) provided yearly training for all personnel about MTP deployment. The analysis identified an association between delayed MTP deactivations and waste (RR = 1.48, CI 1.19–1.85, *p* = 0.0005). An overall improvement in waste was seen over the years, but this could not be attributed to increased closed-loop communication as determined by the proportion of non-stop activations (F(124,3) = 0.98, not significant). Delayed MTP deactivations are the primary determinant of blood component waste. Our proactive intervention on communications between groups was not sufficient in reducing the number of delayed deactivations. However, implementing a digital timeline and regular repetitive training yielded a significant reduction in wasted blood components.

## 1. Introduction

Exsanguinating hemorrhage is a leading cause of death following trauma, accounting for up to a quarter of all deaths among trauma patients [1,2]. Many trauma centers have instituted programs designed to detect and treat post-traumatic hemorrhage to reduce this burden promptly. Specifically, massive transfusion protocols (MTPs) constitute a series of predetermined steps that healthcare providers may activate when treating patients who require large volumes of blood components. MTPs are widely used in United States trauma practice. They have been developed to provide early and standardized blood component delivery in a predetermined ratio to improve patient outcomes while controlling waste [3,4,5].

Optimal implementation of an MTP calls for periodic evaluation and revisions. The American College of Surgeons (ACS) Trauma Quality Improvement Program (TQIP) has published general guidelines regarding performance indicators involved in developing and assessing an MTP that include timely activation, protocol adherence, and proper termination [6]. MTPs should be regularly updated according to the newest guidelines and confirmed published data to ensure best practice in treating exsanguinating trauma patients. Furthermore, MTPs should be frequently evaluated to ensure the protocol meets objectives and identifies potential improvement areas.

Blood components are administered in a predetermined ratio in the early phase of trauma resuscitation to achieve hemostasis in exsanguinating trauma patients [7,8]. Consequently, during MTPs, large amounts of packed red blood cell (PRBC), fresh frozen plasma (FFP), and platelet (PLT) units are continuously prepared by the blood bank and made available for rapid administration in predetermined ratios. Because large amounts of blood components are made available, significant units are discarded, whether due to expiration, contamination, or premature thawing. In a recent study, Roberts et al. modeled global blood component supply and demand, finding that high-income nations have adequate blood stocks to meet their needs, but middle- and low-income countries in South America and Eastern/Central Europe do not [9]. However, reducing blood product waste is an appropriate goal for all nations since it could preserve useful resources and reduce overall healthcare costs.

We hypothesize that blood component waste during MTP can be reduced by analyzing the different elements of MTP, including time indicators and other possible sources of waste. We also hypothesize that communication problems during MTP contribute to the excess of available blood components, leading to significant waste when not administered in a timely manner or adequately returned. We present an analysis of four years of MTP activations at our American College of Surgeons-accredited, Level 1 trauma center, and we identify potential sources of blood component wastage.

## 2. Materials and Methods

This project was generated from an internal quality improvement (Q.I.) initiative undertaken by the Department of Surgery of the University of South Florida, Division of Trauma, at Tampa General Hospital (TGH) (Tampa, FL, USA), an ACS-accredited, Level 1 trauma center. The analysis regarded MTP use and consisted of three different processes: (1) development of the assessment instrument, (2) data analysis, and (3) implementation of a Q.I. initiative.

### 2.1. Development of the Assessment Tool for MTP

A standardized data collection instrument was developed in August 2018 to assess blood component wastage during MTP activations. Based on a previous instrument used by Bawazeer et al. [10], the assessment tool took into account crucial steps of these authors’ institutional MTP and 3 out of 5 performance indicators proposed by ACS TQIP [6]. It consisted of the following three sections:

1. Collection and description of general data and demographics of patients, comprehensive Abbreviated Injury Scale (AIS), Injury Severity Score (ISS), mechanism of traumatic injury, and mortality during admission;
2. Number of blood component units issued, transfused, returned, and discarded; and
3. Proper sequence of MTP activation (defined as a request to the blood bank within 15 min from the clinical decision) and termination (transmitted within 60 min of the clinical decision).

A panel of three senior physicians (one anesthesiologist and two trauma surgeons) plus members of the trauma committee comprising operating room and blood bank personnel reviewed the instrument’s content for accuracy. They provided appropriate modifications to ensure validity. The instrument is available as an Online Supplement (Table S1).

### 2.2. Data Collection and Analysis

A retrospective cohort analysis was performed using the assessment tool. Adult trauma patients who had an activated MTP between 1 January 2015, and 31 December 2018, were eligible. During the study period, institutional criteria for MTP activation were trauma plus one of the following criteria: shock, active bleeding, or immediate transfusion requirements as clinically determined by the provider (emergency medicine attending, trauma surgeon, or anesthesiologist). Patients aged < 18 years, pregnant women, and patients transferred from another facility were excluded from the study.

The primary outcome of this study was to measure blood component wastage during MTP activations. Wastage was defined as (i) a blood component that was obtained after MTP activation but not administered and eventually discarded, (ii) a blood component that was neither administered to the patient nor returned to the blood bank, or (iii) a blood product that was returned to the blood bank but was considered unsuitable for transfusion (e.g., expired or not temperature controlled).

Data were extracted manually by investigators from individual patient charts and provided through a blood bank request. All data obtained were subsequently coded onto a master sheet using a Microsoft Office Excel spreadsheet (Version 2016, Microsoft Corporation, Redmond, WA, USA). A separate investigator performed weekly data monitoring, and any discrepancies between the patient chart or blood bank data and the master sheet were subsequently corrected. The acquisition costs of wasted blood components were calculated using blood bank reported annual per-unit costs multiplied by the number of units wasted; costs are reported in United States dollars (USD) (Online Supplement: Table S2). The data are available from the corresponding authors upon reasonable request.

### 2.3. Quality Improvement Interventions

In November 2016, a multidisciplinary team was created to reduce hospital blood component wastage. This team included representatives from the five departments involved in trauma MTP activations: emergency department, trauma surgery, anesthesiology, intensive care unit, and blood bank. The team formed a blood utilization trauma committee, which began holding weekly meetings to discuss all MTP activations that occurred during the previous week with a specific focus on blood component usage and wastage. Three primary quality improvement interventions resulted from these meetings. First, a communication channel between all five departments was implemented by establishing quarterly meetings to discuss potential improvement areas. In addition, reciprocal agreements found the need for closed-loop communications, especially during MTP activations and deactivations. The personnel was then asked to start giving verbal confirmation feedback of verbal message reception to avoid misunderstandings when communicating between team members or different teams. This intervention was designed to reduce blood component wastage due to delayed communication between the clinical team and the blood bank. Second, the blood bank created specific digital timelines for every MTP activation detailing when and how many units of each blood component were issued, transfused, returned, and discarded. These timelines were made available to all involved personnel. Third and finally, all MTP personnel received additional education on proper MTP activation, deployment, and deactivation. During departmental meetings, these training sessions occurred and emphasized the concepts of blood component handling and cold chain preservation. These last two interventions were designed to increase awareness of MTP waste among providers.

### 2.4. Ethical Considerations

After the first year of evaluation, this project was also submitted and approved by the local institutional review board (USF 2019/37677) for retrospective data analysis. Confidentiality of information was maintained, and all data were collected and de-identified.

### 2.5. Statistical Analysis

Kolmogorov–Smirnov testing was utilized to determine the distribution of each data set. The distributions of several continuous variables were found to be significantly skewed (*p* < 0.05); therefore, median and interquartile range (IQR) were utilized as a measure of central tendency and variance, respectively. Furthermore, nonparametric tests were selected for analysis. Qualitative endpoints such as patients’ demographic characteristics were analyzed descriptively through their distribution frequency utilizing Kruskal–Wallis and Fischer’s Exact where appropriate. To determine interactions between year-to-year effects of the Q.I. initiative and deactivation classification, an aligned rank transformation (ART) multiple factor analysis of variance (ANOVA) was selected. Simple effects were isolated using one-way ART ANOVA. Tukey’s honestly significant difference (HSD) test was selected for post hoc analysis. All data were analyzed using IBM SPSS Statistics for Windows, Version 25.0 (IBM Corp. Released 2017. Armonk, NY). Significance was determined as *p* < 0.05.

## 3. Results

A total of 134 MTP activations in the same number of patients were available during the selected period. Two patients were pregnant, three patients were under 18 years old, four patients were transferred from another facility while receiving MTP, and one activation was a mistake. It was recalled before any blood components were issued. Excluding those 10 listed, a total of 124 adult MTP activations were included in the present analysis.

### 3.1. General Characteristics of Patients

In concordance with current trauma epidemiology, most of our patients were male and reported blunt trauma [11]. As shown in Table 1, the included subjects ranged from 18 to 99 years old (median = 38 years old), and 77% were male. Most of the patients (68%) suffered from blunt trauma injuries, with a median (IQR) ISS of 29 (21–41). Chest AIS was the most reported (78%), followed by abdominal AIS (71%). More than half of the patients (57%) died during admission (Table 1).

### 3.2. Wastage

From 2015–2018, a total of 3668 PRBC units, 2074 FFP units, and 717 PLT units were released from the blood bank. Of the 124 MTP activations, 56% were deactivated within 60 min of the order. Therefore, 44% were classified as nonstop activations, meaning a clinical decision to terminate MTP was ordered but not communicated within 60 min to the blood bank (Table 2).

**Figure 1:**
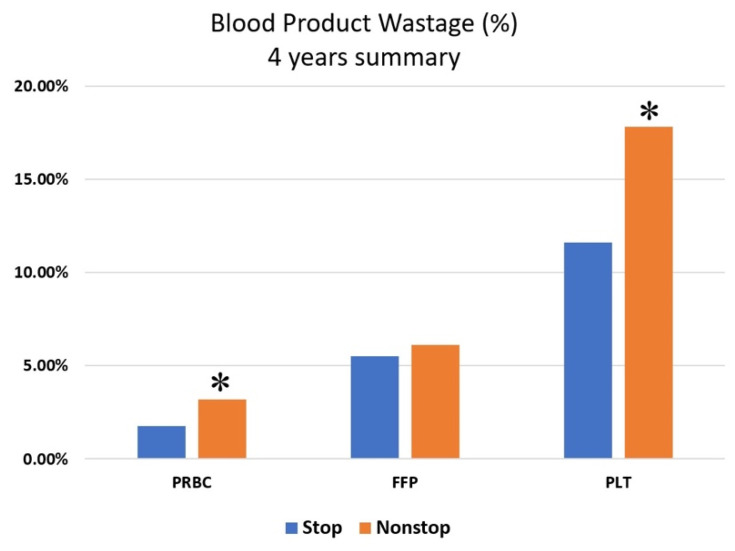
Average % wastage of issued blood components over the four years for normal-stop and nonstop MTP activations. The relative risk of wastage was calculated for individual blood components. Nonstop activations were associated with a higher proportion of wastage in Packed Red Blood Cells (PRBCs) (RR = 1.83, CI 1.19–2.81, *p* = 0.006) and Platelets (PLTs) (RR = 1.55, CI 1.07–2.23, *p* = 0.02); the association was not significant in Fresh Frozen Plasma (FFP) (RR = 1.20, CI 0.84–1.71, N.S.). *: *p* < 0.05.

Interestingly, while nonstop activations were associated with a higher incidence of wastage in PRBCs (RR = 1.8329, CI 1.1989–2.8134, *p* = 0.006) and PLTs (RR = 1.55, CI 1.07–2.23, *p* = 0.02), this reduction was not significant in FFP (RR = 1.20, CI 0.84–1.71, N.S.) (Figure 1).

The Q.I. initiative’s first aim was to improve interdepartmental communication between providers and the blood bank to reduce wastage resulting from delayed termination of MTPs. However, the analysis did not show improvement in the number of improperly terminated MTP activations over the four years since all nonstop terminations remained over 50% during each of the four years. One-way ANOVA confirmed this between the four years (F(124,3) = 0.98, N.S.).

Nevertheless, there was a significant reduction in mean blood component wastage of PRBCs (F(3,124) = 3.841, *p* = 0.011) and PLTs (F(3,124) = 10.71, *p* = 0.0001) in the years 2017–2018 after quality improvement initiatives were implemented after November 2016. However, no significant changes were observed in the mean wastage of FFP. The improvement in PRBC and PLT wastage supports the efficacy of the two interventions designed to increase MTP waste awareness: creating timelines of MTP activations by the blood bank and additional continuing personnel training.

## 4. Discussion

This project summarizes four years of MTP activations at our Level 1 trauma center in central Florida. The latter two years involved implementing an internal quality improvement initiative by analyzing major trauma MTP activations. Among the 124 activations analyzed, a total of 3668 PRBC, 2074 FFP, and 717 PLT units were issued from the blood bank, which was an average of 29.6 PRBC, 16.7 FFP, and 5.8 PLT units per patient. Considering that more blood components are issued than are transfused, these numbers are in the same range as those reported in the PROPPR trial [8].

Blood component waste during MTPs is underreported in the current literature. Dunbar et al. found that their center routinely had an unacceptably high waste rate—up to 61% of blood products issued—after MTP activations in both trauma and non-trauma patients [12]. Within our analyzed period, we found a similar proportion of waste. However, the mean wastage of PLTs and PRBCs decreased after the implementation of several Q.I. interventions. Notably, PRBC wastage decreased below the maximum threshold recommended by the American Association of Blood Banks: 5% of components issued [13]. However, a significant reduction in FFP was not observed: This may result from the short half-life of FFP when removed from refrigeration, preventing it from being returned in non-usage. It is worth mentioning that the overall improvement in blood component wastage contributed to a significant reduction in wastage-related costs, from an estimated total of USD 32,021.73 in 2015 to USD 5654.50 in 2018 (Online Supplement: Table S2).

In the first two years of our review, the wastage of PLTs was much higher than the wastage of other blood components. This issue could be related to their non-usage during the first resuscitation phase, meaning PLT units were delivered by the blood bank but not immediately transfused. Similarly, the non-significant difference in FFP wastage could be related to the inherent fragility of FFP, which cannot be saved after thawing and must be discarded, even if unused. Indeed, the literature describes that plasma wastage increases when an inventory of thawed plasma is made readily available [14].

More than half of the MTP activations were properly terminated within 1 h (Table 2). This result is in the same range as Bawazeer et al., who reported that MTP was deactivated within 1 h in 50% of cases [10], which is significantly lower than the 92% rapid rate deactivations described by Cotton et al. [15]. The notable differences could explain this variability among MTPs of each facility since each protocol should be adapted to local resources, blood bank policies, and trauma system organization [5,16].

In comparison to properly terminated activations, nonstop MTP activations were associated with increased waste of blood components (Figure 1), reaching statistical significance for PRBC and PLT units. This finding suggests that a primary determinant of blood component wastage is inefficient communication with the blood bank or, more specifically, a delayed notification that MTP has been deactivated. Communication failure among medical team members is a recognized problem and contributes to most sentinel events [17]. Clear communication with the blood bank is fundamental to ensuring timely preparation and delivery of safe blood components. When the decision to terminate MTP is reached, the blood bank must be promptly notified to stop the setup of additional blood components.

In this vein, one of the Q.I. initiatives was designed to increase interdepartmental communication between providers and the blood bank and decrease nonstop activations. Although the frequency of MTP nonstop activations did not improve, we did observe a decrease in mean PLT and PRBC wastage in subsequent years of the Q.I. initiative. Currently, no endpoints adequately explain this observed phenomenon. Likely, our interventions of weekly departmental meetings, the introduction of digital timelines of blood components, and annual re-training made providers more waste conscious. However, we did not measure the “waste consciousness” of providers. In the future, similar Q.I. initiatives could track practitioners’ awareness of the problem with a self-assessment tool.

Both initiation and termination are critical steps of an MTP deployment [18]. From a Q.I. perspective, there should be a focus on the accuracy of decision tools and clinical judgment and time needed to perform these tasks. According to our results, in only 69% of cases was the activation order received and initiated by the blood bank within 15 min. Similarly, we found a low rate (44%) of timely MTP deactivation within 60 min from the order. A possible explanation is an inadequate logistics and communications network within our facility. When managing a massively bleeding trauma patient, efficient logistics and proper communication protocols maintain a fundamental role in reducing delivery errors, deterioration of blood components, and delivery time and administration time. Making blood components readily available has demonstrated an association with low blood usage rates and low mortality [19]. Apart from traditional interventions, periodic personnel training through in-situ simulations was recently proposed to promote technical and non-technical skills during MTP activations [20]. Unfortunately, our intervention designed to facilitate communications failed to meet expectations. In the future, a more granular analysis can be conducted with additional interventions focusing on communication. For example, a periodic notification could ask practitioners if the MTP should be stopped, or written checklists in operating rooms could be updated to include this reminder during timeouts.

Several limitations to this project need to be acknowledged. Firstly, this work was limited in scope. Due to the substantial differences in local MTP and other institutional protocols, the results of this single-center quality evaluation may not generalize to other Level 1 trauma centers. Confounders such as human factors and interactions with other internal protocols were not analyzed, adding potential error sources. Furthermore, even though the data collection tool was internally validated and based on expert consensus, it was never tested for reliability. Data were retrospectively collected, potentially resulting in unknown confounders and gaps in data collection. Previous MTP evaluations were made through retrospective data analysis, but a standard evaluation tool has yet to be established within the literature [21,22,23]. A standardized tool could easily prompt periodic MTP assessments and provide standardized data to allow more precise benchmarks across regional and national trauma networks. A possible future application of this MTP assessment tool could be a rigorous analysis of the adherence impact on outcomes based on larger patient samples.

## 5. Conclusions

This work offers a detailed and individualized perspective of MTP activations and could be used as a resource for trauma centers seeking to improve their MTP and reduce waste. One of the more significant findings of this work is a relationship between nonstop MTP activations and blood component waste, especially PRBCs and PLTs.

To reduce waste, hospitals should collaborate with blood banks to track the release and return of blood components, facilitate periodic re-training of involved personnel, and ameliorate communications between providers and between departments during activations.

## Figures and Tables

**Table 1:** General features of the 124 patients who had an activated massive transfusion protocol at Tampa General Hospital between 2015 and 2018.

Criteria	Subgroup	Results
Age, range; median (IQR)		18–99; 38 (27–53) years
Gender, n (%)	Male	96 (77%)
ISS range; median (IQR)		9–75; 29 (21–41)
Mechanism of injury, n (%)		
	Blunt	84 (68%)
	Penetrating	40 (33%)
Head and spine AIS, n (%)		65 (52% out of the total)
	≤3	23 (35%)
	>3	42 (65%)
Chest AIS, n (%)		97 (78% out of the total)
	≤3	56 (58%)
	>3	41 (42%)
Abdomen AIS, n (%)		88 (71% out of the total)
	≤3	49 (56%)
	>3	39 (44%)
Mortality during admission, n (%)		70 (57%)

The study participants were mostly male, relatively young (median age: 38 years), and mainly suffering from blunt trauma injuries. IQR: interquartile range; ISS: Injury Severity Score; AIS: Abbreviated Injury Scale.

**Table 2:** Timely activation and termination of MTP.

Criteria	% Compliance	Comments
MTP activation followed indications	81%	Activation criteria: shock OR active bleeding OR immediate need of transfusion.
MTP activation occurred promptly	69%	Within 15 min of a doctor’s decision
Timely MTP termination	56%	Within 60 min of deactivation order

MTPs were promptly activated 69% of the times and were terminated within 60 min of deactivation order in 56% of cases MTP: massive transfusion protocol.

## Data Availability

The data are available from the corresponding authors upon reasonable request.

## Supplementary Materials

The following are available online at https://www.mdpi.com/1660-4601/18/1/274/s1, Table S1: Massive transfusion protocol self-assessment instrument, Table S2: Cost analysis.

## References

[1] Pfeifer R., Tarkin I.S., Rocos B., Pape H.C. (2009). Patterns of mortality and causes of death in polytrauma patients--has anything changed?. Injury.

[2] Hi T.J., Mohamad Y., Lip H.T.C., Noh N.M., Alwi R.I. (2017). Prognostic predictors of early mortality from exsanguination in adult trauma: A Malaysian trauma center experience. Trauma Surg. Acute Care Open..

[3] Balvers K., Coppens M., Van Dieren S., van Rooyen-Schreurs I.H., Klinkspoor H.J., Zeerleder S.S., Baumann H.M., Goslings J.C., Juffermans N.P. (2015). Effects of a hospital-wide introduction of a massive transfusion protocol on blood product ratio and blood product waste. J. Emerg. Trauma Shock.

[4] Chuster K.M., Davis K.A., Lui F.Y., Maerz L.L., Kaplan L.J. (2010). The status of massive transfusion protocols in United States trauma centers: Massive transfusion or massive confusion?. Transfusion.

[5] Treml A.B., Gorlin J.B., Dutton R.P., Scavone B.M. (2017). Massive transfusion protocols: A survey of academic medical centers in the United States. Anesth. Analg..

[6] ACS TQIP Best Practices Guidelines in Massive Transfusion in Trauma, Technical Report, 2014. Retrieved 1 August 2018. https://www.facs.org/quality-programs/trauma/tqp/center-programs/tqip/best-practice.

[7] Holcomb J.B., Del Junco D.J., Fox E.E., Wade C.E., Cohen M.J., Schreiber M.A., Alarcon L.H., Bai Y., Brasel K.J., Bulger E.M. (2013). The prospective, observational, multicenter, major trauma transfusion (PROMOTE) study: Comparative effectiveness of a time-varying treatment with competing risks. JAMA Surg..

[8] Holcomb J.B., Tilley B.C., Baraniuk S., Fox E.E., Wade C.E., Podbielski J.M., del Junco D.J., Brasel K.J., Bulger E.M., Callcut R.A. (2015). Transfusion of plasma, platelets, and red blood cells in a 1:1:1 vs. a 1:1:2 ratio and mortality in patients with severe trauma: The PROPPR randomized clinical trial. JAMA.

[9] Oberts N., James S., Delaney M., Alsharif U. (2019). The global need and availability of blood products: A modelling study. Lancet Haematol..

[10] Bawazeer M., Ahmed N., Izadi H., McFarlan A., Nathens A.B., Pavenski K. (2015). Compliance with a massive transfusion protocol (MTP) impacts patient outcome. Injury.

[11] Cothren C.C., Moore E.E., Hedegaard H.B., Meng K. (2007). Epidemiology of urban trauma deaths: A comprehensive reassessment 10 years later. World J. Surg..

[12] Dunbar N.M., Olson N.J., Szczepiorkowski Z.M., Eric M., Tysarcyk R.M., Triulzi D.J., Alarcon L.H., Yazer M.H. (2016). Blood component transfusion and wastage rates in the setting of massive transfusion in three regional trauma centers. Transfusion.

[13] Hannon T. (2015). Waste Not, Want Not. Am. J. Clin. Pathol..

[14] Novak D.J., Bai Y., Cooke R.K., Marques M.B., Fontaine M.J., Gottschall J.L., Carey P.M., Scanlan R.M., Fiebig E.W., Shulman I.A. (2015). Making thawed universal donor plasma available rapidly for massively bleeding trauma patients: Experience from the Pragmatic, Randomized Optimal Platelets and Plasma Ratios (PROPPR) trial. Transfusion.

[15] Cotton B.A., Dossett L.A., Au B.K., Nunez T.C., Robertson A.M., Young P.P. (2009). Room for (performance) improvement: Provider-related factors associated with poor outcomes in massive transfusion. J. Trauma.

[16] Etchill E., Sperry J., Zuckerbraun B., Alarcon L., Brown J., Schuster K., Kaplan L., Piper G., Peitzman A.B., Neal M.D. (2016). The confusion continues: Results from an American Association for the Surgery of Trauma survey on massive transfusion practices among United States trauma centers. Transfusion.

[17] Lingard L., Espin S., Whyte S., Regehr G., Baker G.R., Reznick R., Bohnen J., Orser B., Doran D., Grober E. (2004). Communication failures in the operating room: An observational classification of recurrent types and effects. Qual. Saf. Health Care.

[18] Foster J.C., Sappenfield J.W., Smith R.S., Kiley S.P. (2017). Initiation and Termination of Massive Transfusion Protocols: Current Strategies and Future Prospects. Anesth. Analg..

[19] Hess J.R., Ramos P.J., Sen N.E., Cruz-Cody V.G., Tuott E.E., Louzon M.J., Bulger E.M., Arbabi S., Pagano M.B., Metcalf R.A. (2017). Quality management of a massive transfusion protocol. Transfusion.

[20] Langston A., Downing D., Packard J., Kopulos M., Burcie S., Martin K., Lewis B. (2017). Massive Transfusion Protocol Simulation: An Innovative Approach to Team Training. Crit. Care Nurs. Clin. N. Am..

[21] Ogrinc G., Davies L., Goodman D., Batalden P., Davidoff F., Stevens D. (2016). SQUIRE 2.0 (Standards for Quality Improvement Reporting Excellence): Revised publication guidelines from a detailed consensus process. BMJ Qual. Saf..

[22] Nunez T.C., Young P.P., Holcomb J.B., Cotton B.A. (2010). Creation, Implementation, and Maturation of a Massive Transfusion Protocol for the Exsanguinating Trauma Patient. J. Trauma.

[23] Batalden P.B., Davidoff F. (2007). What is “quality improvement” and how can it transform healthcare?. Qual. Saf. Health Care.

