# Supplementary material for: Quality Improvement Project of a Massive Transfusion Protocol (MTP) to Reduce Wastage of Blood Components
Source: Int J Environ Res Public Health. 2021 Jan 1;18(1):274. doi: 10.3390/ijerph18010274 (PMC7795105; doi:10.3390/ijerph18010274)
Supplement: Supplementary file 1 [file ijerph-18-00274-s001.pdf]

Supplementary

Table S1. Massive Transfusion Protocol self-assessment instrument.

**1) Demographics and trauma characteristics**	
Age	years
Gender	Male / female
ISS	
Mechanism of Injury	Blunt / Penetrating
ED SBP	
ED GCS	
Head AIS	≤ 3 / > 3
Chest AIS	≤ 3 / > 3
Abdomen AIS	≤ 3 / > 3
Survival during admission	YES/NO
**2) Wastage**	
Products issued	By type (PRBC; FFP; PLT)
Products infused	By type (PRBC; FFP; PLT)
Products wasted	By type (PRBC; FFP; PLT)
**3) MTP key actions and quality indicators**	
MTP activation followed indications	Activation criteria: shock OR active bleeding OR immediate need of transfusion.
MTP activation occurred promptly	within 15 minutes of doctor's decision
Group and screen sent	From trauma bay
Hemorrhage panel sent	From trauma bay
Blood products administered in a predetermined ratio	According to the MTP protocol of the facility, the ratio was 6:4:1 (PRBC:FFP:PLT)
Tranexamic acid	First dose within 3 hours
ABG measured	Within 30 minutes of MTP activation
Lactate measured	Within 30 minutes of MTP activation
Fibrinogen measured	During MTP activation
CBC measured	Before and during MTP activation
Electrolytes measured	Before and during MTP activation
Ca^{++} re-check	Re-check within 2 hours of MTP activation
K^{+} re-check	Re-check within 2 hours of MTP activation
rTEG used	As per protocol – after the first round of blood products
Timely MTP termination	Within 60 minutes of deactivation order

ISS: Injury Severity Score; ED: Emergency Department; SBP: Systolic Blood Pressure; GCS: Glasgow Coma Scale; AIS: Abbreviated Injury Scale; ICU: Intensive Care Unit; PRBC: Packed Red Blood Cells; FFP: Fresh Frozen Plasma; PLT: platelets; MTP: Massive Transfusion Protocol; ABG: Arterial Blood Gas; CBC: Complete Blood Count; rTEG: rapid Thromboelastography.

Table S2. Cost Analysis.

Institutional cost of blood products 2015-2016	
**Blood Product**	**Cost (USD)**
Packed Red Blood Cells	$183.31
Fresh Frozen Plasma	$55.29
Platelets	$504.40
**Institutional cost of blood products 2017-2018**	
**Blood Product**	**Cost (USD)**
Packed Red Blood Cells	$196.50
Fresh Frozen Plasma	$57.00
Platelets	$520.00

Year	Blood Product Wasted	Wastage Costs
2015	Packed Red Blood Cells: 44	$8,065.64
	Fresh Frozen Plasma: 41	$2,266.89
	Platelets: 43	$21,689.20
	Total	$32,021.73
2016	Packed Red Blood Cells: 33	$6,049.23
	Fresh Frozen Plasma: 48	$2,653.92
	Platelets: 45	$22,698.00
	Total	$31,401.15
2017	Packed Red Blood Cells: 1	$196.50
	Fresh Frozen Plasma: 5	$285.00
	Platelets: 3	$1,560.00
	Total	$2,041.50
2018	Packed Red Blood Cells: 3	$589.50
	Fresh Frozen Plasma: 25	$1,425.00
	Platelets: 7	$3,640.00
	Total	$5,654.50
